# Efficient classification of COVID-19 CT scans by using q-transform model for feature extraction

**DOI:** 10.7717/peerj-cs.553

**Published:** 2021-06-15

**Authors:** Razi J. Al-Azawi, Nadia M.G. Al-Saidi, Hamid A. Jalab, Hasan Kahtan, Rabha W. Ibrahim

**Affiliations:** 1 Department of Laser and Optoelectronics Engineering, University of Technology, University of Technology, Baghdad, Iraq, Iraq; 2 Department of Applied Sciences, University of Technology, University of Technology, Baghdad, Iraq, Iraq; 3 Department of Computer System and Technology, Faculty of Computer Science and Information Technology, University of Malaya, Kuala Lumpur, Malaysia; 4 Department of Software Engineering, Faculty of Computer Science and Information Technology, University of Malaya, Kuala Lumpur, Malaysia; 5 IEEE: 94086547, Kuala Lumpur, Malaysia

**Keywords:** COVID-19, Machine learning, q-transform, CT scans, Classification, Support vector machine, k-nearest neighbor, Feature extraction, Features reduction

## Abstract

The exponential growth in computer technology throughout the past two decades has facilitated the development of advanced image analysis techniques which aid the field of medical imaging. CT is a widely used medical screening method used to obtain high resolution images of the human body. CT has been proven useful in the screening of the virus that is responsible for the COVID-19 pandemic by allowing physicians to rule out suspected infections based on the appearance of the lungs from the CT scan. Based on this, we hereby propose an intelligent yet efficient CT scan-based COVID-19 classification algorithm that is able to discriminate negative from positive cases by evaluating the appearance of lungs. The algorithm is comprised of four main steps: preprocessing, features extraction, features reduction, and classification. In preprocessing, we employ the contrast limited adaptive histogram equalization (CLAHE) to adjust the contrast of the image to enhance the details of the input image. We then apply the q-transform method to extract features from the CT scan. This method measures the grey level intensity of the pixels which reflects the features of the image. In the feature reduction step, we measure the mean, skewness and standard deviation to reduce overhead and improve the efficiency of the algorithm. Finally, “k-nearest neighbor”, “decision tree”, and “support vector machine” are used as classifiers to classify the cases. The experimental results show accuracy rates of 98%, 98%, and 98.25% for each of the classifiers, respectively. It is therefore concluded that the proposed method is efficient, accurate, and flexible. Overall, we are confident that the proposed algorithm is capable of achieving a high classification accuracy under different scenarios, which makes it suitable for implementation in real-world applications.

## Introduction

The rapid spread of “coronavirus SARS-CoV-2” during the first few months of 2020 has led the World Health Organization (WHO) to classify the disease as a pandemic. The total reported number of cases for COVID-19 has exponentially increased from only 282, as of 21 January 2020, to 11 million, as of February 2021 (World Health Organization, 2021). With such a high number of cases, it is imperative to find ways to reduce the burden of diagnosis on the healthcare systems. One of such ways is to help clinicians make faster yet accurate decisions. COVID-19 is routinely diagnosed using a number of methods, one of which is through the use of computed tomography (CT) scan. CT is a non-invasive imaging technology used to obtain high quality images of the body for the diagnosis or monitoring of several diseases (Hasan et al., 2020). The CT imaging utilizes X-ray radiation to quantify the density of the target tissue. Highly-dense tissues, such as bones, absorb greater amounts of radiation, thereby exhibiting stronger signal intensity. In contrast, softer tissues, such as lungs, absorb less radiation and exhibit weaker signals. Accordingly, it is not always feasible to clearly distinguish the boundaries between different types of tissues and organs. The SARS-CoV-2 mainly attacks the lungs. This causes the formation of fibrotic tissue which can be detected through CT imaging (World Health Organization, 2021). Lung fibrosis involves the scarring and stiffening of the lung tissue, thereby giving the tissue a dense appearance in the CT scan.

Generally, the majority of the image classification algorithms use either handcrafted or deep learning for features extraction. In the handcrafted model, the primary features such as shape and texture are extracted. While the deep learning algorithm extracts features from images through the convolutional layers (Ahmad, Farooq & Ghani, 2021). Recent work presents some benefits of using the deep learning CNN as a feature extraction (Bhattacharya et al., 2021). However, the limited amount of patient data and the computational load are inevitable challenges for the training of CNN (Gadekallu et al., 2020). To overcome this limitation, there are methods that work on the basis of combination of handcrafted and deep learning CNN features extraction (Hasan et al., 2019). However, these techniques require high hardware capacity and balanced image dataset. This can lead to poor performance, not only when using the dataset with CNN but also the classification of features with other classifiers (Vasan et al., 2020). This observation motivated us to ensure that the extracted features should represent the image characteristics that are related to the classification.

Unlike the existing techniques of machine learning with large number of extracted features, the proposed work relays on the new q-transform model to extract the optimal features from the CT scans.

Based on this, we propose in this study an automated algorithm for the classification of COVID-19 cases based on the appearance of lungs in CT scans. The fully-automated computer algorithm in the clinical setting could reduce the diagnosis time and improve the workflow at the medical facility. This could ultimately alleviate the burden on the healthcare system. The proposed algorithm comprises four primary steps: preprocessing, features extraction, features reduction, and classification. In the first step, the contrast of the input image is adjusted using CLAHE to enhance the details of the input image. This helps improve the overall accuracy of the algorithm by allowing features to be extracted properly. In the second and third steps, the q-transform method is used to extract only the essential features from the image. Finally, a number of classifiers are used for the classification process. Moreover, 5-fold cross validation is employed to determine whether an input image is healthy (i.e., not infected) or pathological (i.e., infected). These classifiers include the “k-nearest neighbor”, “decision tree”, and “support vector machine”. Judging by the obtained experimental results, the proposed algorithm is accurate and efficient, and can be considered a suitable algorithm to be implemented in real-world applications and clinical settings.

## Related work

The process of image classification perquisites the extraction of visual features from input images. When considering the features extraction methods, the reported image classification algorithms can be classified under two main categories: handcrafted, and deep learning. In the handcrafted model, the shape and texture comprise the primary features that are extracted from medical images for the purpose of classification (Olayemi, Zare & Fermi, 2019; Venkatraman & Alazab, 2018). These features are either extracted from the whole image or from subregions within the images to obtain a large set of features that represents the input image (Dimitrovski et al., 2015; Kumar et al., 2016). In that sense, the input image is represented by a set of features which depict certain characteristics of the image, such as textures, colors, shapes, and patterns.

Öztürk, Özkaya & Barstuğan (2021) proposed a low dimension handcrafted features for image classification to detect COVID-19 in X-ray and CT images. The extracted features were based on GLCM; LBGLCM; GLRLM, and SFTA. The achieved classification accuracy was 98%. The good classification performance was due to the limited testing image samples used.

Correspondingly, the type and quality of the extracted features define the robustness of methods that use the handcrafted model. This constitutes the primary limitation of the handcrafted model, since some essential features may be overlooked while non-essential counterparts are over-represented. To mitigate this, several works have been proposed to automate the classification of COVID-19 using deep learning with handcrafted model as feature extraction.

Zhang et al. (2021) proposed a combination of deep convolutional and handcrafted features extracted from X-ray chest scans. The extracted features included the GTexture; the Gray-Level Co-Occurrence Matrix (GLCM); the Gray Level Difference Method (GLDM); the Fast Fourier Transform, Wavelet transform, and Local Binary Pattern. The combining of features has improved the performance of the classification task up to 98.8%. This method used a large number of features and relays on both image content and image quality.

In another work, Hasan et al. (2020) proposed an algorithm which combines deep learning with q-deformed entropy for feature extraction to classify lung CT scans as COVID-19, pneumonia, or healthy cases. The reported SVM classification accuracy was 96.20% for a collected dataset comprising 321 lung CT scans. However, the feature extraction using deep learning approach needs large datasets to be capable of distinguishing between COVID-19 and other infectious viral diseases. Moreover, Shankar & Perumal (2020) proposed a novel fusion model hand-crafted with deep learning features model for diagnosis and classification of COVID-19. The feature extraction model incorporated the fusion of handcrafted features with the help of local binary patterns and deep learning features. The achieved accuracy was 94.85% on a dataset composed of 27 images under normal class, 220 images under COVID-19, 11 images under SARS and 15 images in Pneumocystis class. The limitation of this method is associated with the small training dataset.

Methods implementing CNNs demonstrate high classification accuracy and better overall performance compared to their handcrafted counterparts (Bhattacharya et al., 2021). However, despite their advantages, CNNs may sometimes extract inadequate features from images, which may affect the accuracy of the classification process. To overcome this, a feature selection technique can be used to define essential features for each specific classification application. Hasan et al. (2019) used deep learning to extract features from brain MRI scans. In their work, the handcrafted features were extracted using the “modified gray level co-occurrence matrix” (MGLCM) method to enhance the classification accuracy of the SVM classifier. The reported results demonstrated that the classification accuracy has increased up to 99.30%. This work has demonstrated a novel approach to enhance the overall classification accuracy of deep learning-based algorithms. Li et al. (2020) proposed a CNN-based, fully-automated algorithm that analyzes CT scans to detect COVID-19 cases and differentiate COVID-19 from pneumonia. The reported COVID-19 detection accuracy was 96% for a dataset that comprised 400 CT scans. The work in Song et al. (2020) presented a deep learning-based, automated diagnosis system to identify COVID-19-positive cases through analysis of lung CT scans. The achieved accuracy was 95% when tested with a collected dataset comprising 88, 86 and 100 CT scans of COVID-19, healthy and bacterial pneumonia cases, respectively.

In a recent study (Ahmad, Farooq & Ghani, 2021), a deep COVID-19 classification method has been proposed to distinguish between four different chest-related infections using a large and balanced dataset. The method uses ensemble learning, fine-tuning, data augmentation, and transfer learning. The achieved accuracy was 94%. The limitation of the study was the dataset which was not sufficient for practical COVID-19 identification. The summary of recent studies which deal with the classification of COVID-19 cases from medical images is presented in Table 1.

**Table 1:** Summary of recent studies which deal with the classification of COVID-19 cases from medical images.

Reference	Aim	Dataset	Evaluation	Limitations
Öztürk, Özkaya & Barstuğan (2021)	COVID-19 X-ray and CT scans classification using low dimension features, inlcuding: GLCM, LBGLCM, GLRLM, and SFTA	Chest X-ray and CT scans, with 4 ARds images, 101 COVID-19 images, 2 pneumocystis-pneumonia images, 11 SARS images, and 6 streptococcus images	Achieved a classification accuracy of 98%	Lack of depth analysis using larger datasets. The good classification performance was due to the use of limited samples of testing images
Zhang et al. (2021)	Classification of COVID-19 in chest X-ray scans by using deep convolutional and handcrafted features. The extracted features are: GTexture, GLCM, GLDM, Fast Fourier Transform, Wavelet transform, and LBP	The dataset contained 5,143 X-ray images categorized into COVID-19, normal and pneumonia cases	Achieved a classification accuracy of 98.8%	This method has a large number of features and relays on both image contents and quality which could be inconsistent and subject to artifacts. There were 308 features extracted from each image, and evaluated using 14 different statistical measures. This increases the computational complexity and reduces the efficiency of the method
Hasan et al. (2020)	Classification of COVID-19 cases by using chest CT scans through a combination of Q-Deformed entropy and deep learning features	The dataset comprised 321 chest CT scans, of which 118 were from infected COVID-19 patients, 96 from infected pneumonia patients, and 107 from healthy people	Achieved a classification accuracy of 96.2%	Feature extraction using deep learning approach needs large datasets, as opposed to the small dataset used in their proposed study. Accordingly, more in-depth analyses using larger datasets are needed
Shankar & Perumal (2020)	Classification of COVID-19 cases from chest X-ray images using LBP with deep learning features	The images from the dataset included 27 healthy cases, 220 COVID-19 cases, 11 SARS cases, and 15 Pneumocystis cases	Achieved a classification accuracy of 94.85%	The use of small training dataset may have skewed the classification accuracy of the method
Li et al. (2020)	Classification of COVID-19 cases from volumetric chest CT scans using the deep learning model COVNet	The dataset included 4352 scans from 3322 patients	The model achieved classification sensitivity of 90% and specificity of 96%	Lack of clear methodology for the classification of COVID-19 cases
Song et al. (2020)	Classification of COVID-19 cases from CT scans using deep learning	The images from the dataset included 88 COVID-19 cases, 86 healthy cases, and 100 bacterial pneumonia cases	The achieved accuracy was 95%	The use of deep learning for feature extraction requires large datasets (which is not the case here), in addition to premium hardware

It is noteworthy that most of the datasets employed in earlier COVID-19 image classification studies are imbalanced. Meaning the number of images for different classification categories are not equal (Ahsan et al., 2021). This can introduce bias and reduce the classification accuracy of the method in real life applications. Moreover, other factors, such as feature extraction technique, and the machine-learning algorithm used can play a role in determining the classification accuracy of methods.

From the above, it can be realized that the feature extraction process is the key determinant of the classification accuracy of an algorithm. Accordingly, the extracted features should be representative of the image characteristics and at the same time to improve the performance of the algorithm (Al-Shamasneh et al., 2018; Ala’a et al., 2020; Ibrahim, Hasan & Jalab, 2018; Jalab, Ibrahim & Ahmed, 2017; Jalab et al., 2019). Therefore, in this study, we propose a new and an efficient algorithm to extract essential features from lung CT scans for the classification of COVID-19 cases. The present study achieves the following:

1. Propose an efficient and accurate algorithm for the classification of COVID-19 cases based on lung CT scans using the q-transform algorithm for feature extraction. As well as, using a number of statistical measures to reduce the extracted features to optimal levels in order to improve the overall performance of the algorithm.
2. Test the algorithm with an open-access dataset that contains a large number of CT scans. Such datasets are considered as benchmarks since they are readily available and enable relative comparison with relevant algorithms.
3. Compare the results of the proposed model with the results of a number of recent, state-of-art algorithms that deal with the same topic.

## Materials and Methods

**Figure 1:**
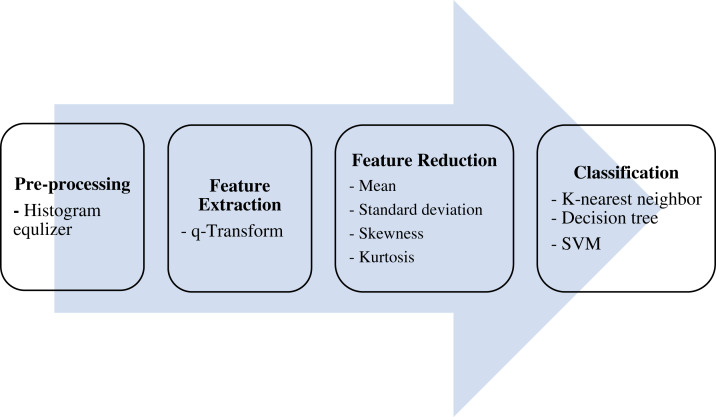
The proposed model.

The ultimate aim of the proposed algorithm is to efficiently classify lung CT scans into either COVID-19 -positive or -negative (healthy) cases. This is done over four main stages: pre-processing, features extraction, features reduction, and classification, as shown in Fig. 1.

### Dataset description

**Figure 2:**
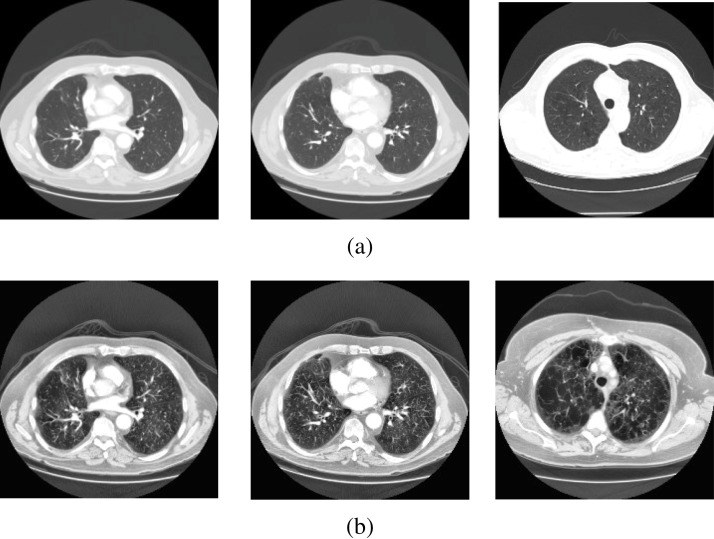
Sample scans from the dataset before and after enhancement showing healthy lungs. (A) Original CT scans, (B) enhanced CT scans.

**Figure 3:**
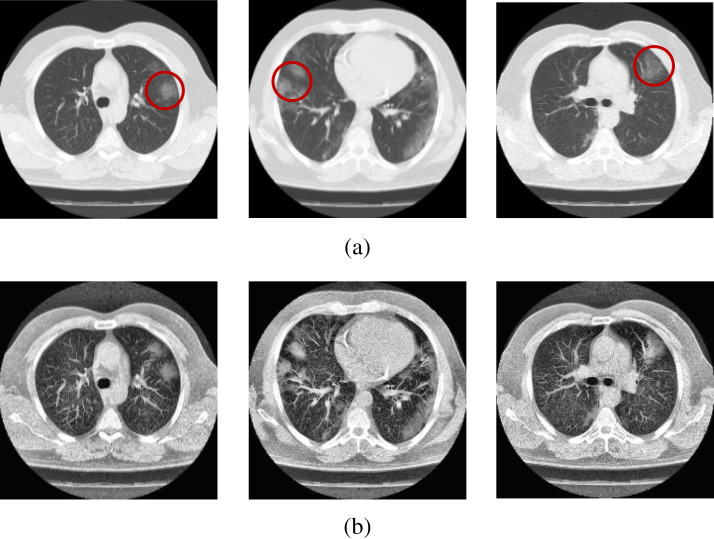
Sample scans from the dataset before and after enhancement showing infected lungs. (A) Original CT scans, with red circles highlighting some regions where fibrosis can be seen; (B) enhanced CT scans.

In this study, lung CT scans dataset was used. The dataset is a balanced dataset and has been obtained from the “Italian Society of Medical and Interventional Radiology (SIRM)” (COVID-19 Database: Casistica Radiologica Italiana, https://www.sirm.org/category/senza-categoria/covid-19/). The dataset comprises 276 lung CT scans which are equally divided into COVID-19 positive and negative cases. The balanced dataset makes the proposed classification model more efficient to provide better prediction accuracy. Sample images from the dataset are presented in Figs. 2 and 3.

### CT lung scan pre-processing

Since CT imaging relies on the measurements provided by several independent detectors, the technology is prone to developing image artifacts. Such artifacts can lead to intensity variation between consecutive scans, and could affect the quality of the scans’ features. To mitigate this issue, a preprocessing step is implemented to facilitate better features extraction and improve the accuracy of the algorithm. Here, we used the “contrast-limited adaptive histogram equalization” (CLAHE) as a preprocessing step for contrast enhancement. By using CLAHE, we are able to accentuate the pixels of the image that represent essential features. CLAHE is based on “Adaptive histogram equalization” (AHE) technique. The contrast of an image is enhanced by computing several histograms, where each histogram corresponds to a region of the image. This allows for local contrast enhancement within an image. However, AHE is prone to over enhance noise under normal circumstances. Thus, CLAHE has been made to overcome this issue by limiting luminance amplification. CLAHE comprises three main steps: generation of tile, equalization of histogram, and bilinear interpolation. Accordingly, the input image is initially split into regions called tiles. Next, histogram equalization is carried out on each of these tiles. The histogram is represented by bins for each tile where bins values that are greater than the clip limit are gathered then distributed to other bins. The output tiles are merged together by using a bilinear interpolation to produce a contrast-enhanced copy of the input image.

### Features extraction

In this study, we propose a new, q-transform-based feature extraction method to extract essential features from lung CT scans for the classification of COVID-19 cases. To improve the performance and efficiency of the algorithm, we extract an optimal number of features from each image (Al-Shamasneh et al., 2018; Ala’a et al., 2020; Jalab, Ibrahim & Ahmed, 2017; Jalab et al., 2019; Roy et al., 2016). As mentioned in the introduction section, COVID-19-induced lung fibrosis can be detected through CT imaging after a few weeks of being infected. This pathological manifestation provides a cue to identify COVID-19 cases from medical images. Therefore, by adopting the deformation theory, we propose DTFE feature extraction model for studying the features of lung tissue from CT scans. The q-derivative ∆qI of a function I(x) is given by Fitouhi & Bettaibi (2006):

(1) $${\Delta _q}I\left( x \right) = \displaystyle{{I\left( {qx} \right) - I\left( X \right)} \over {\left( {q - 1} \right)x}}$$

The q-logarithm deformation is given by Eq. (2) (Umarov, Tsallis & Steinberg, 2008):

(2) $$l{n_q}\left( \chi \right) =\left\{ {\matrix{{\hskip-36pt}{\ln \left( {\rm \chi } \right)\qquad q = 1,\chi > 0} \cr {\displaystyle{{{\chi ^{1 - q}} - 1} \over {1 - q}}\qquad q \ne 1,\; \chi > 0\qquad} \cr {\hskip-26pt}{Undefined\qquad \chi \le 0\qquad} \cr }} \right.$$

The q-transform (Tq) has been intensively explored as a transformation process in various applications, including entropy. Eq. (2) implies the generalization of the following q- transform:

(3) $${T_q}\; \left( x \right) = \; \sqrt { - 2\; l{n_q}\; \left( x \right)\; } {\rm con}\left( {2\pi x} \right)$$

where x is the pixel probability, and q = 0.5 has been chosen experimentally.

To use Tq in feature extraction, we assume a positive number as the value of the pixel, thus, we utilize the radius (|q|) to satisfy this condition. Tq is calculated here according to the intensity of the pixels’ grey level which is representative of the features of the image. In our proposed algorithm, the input image is divided into non-overlapping blocks measuring “w x w” pixels, then Tq is calculated for each of these blocks. From every lung CT scan, a total of 1,024 features are extracted. A summary outlining the steps for the proposed Tq feature extraction model is presented inAlgorithm 1.

**Algorithm 1:** q-transform feature extraction.

Initialization: I=Input image, q=0.5
For each Input image I do
(b1, b2, …, bn) ← divide I into n blocks of size 16 × 16 pixels
For i=1 to n do
Tq ← I=(1,2,…n) // Tq Features of all (n) blocks
EndFor
EndFor

**Figure 4:**
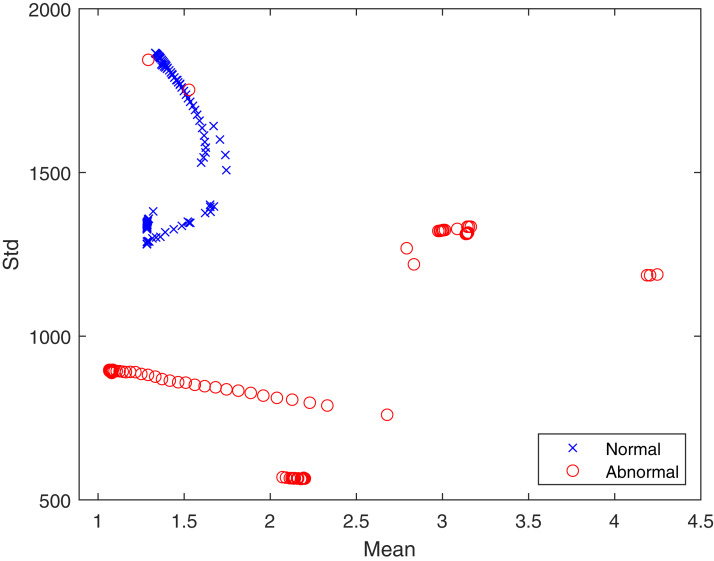
The proposed feature distribution for healthy and infected lungs.

In the proposed feature extraction algorithm, the q transform operator plays a vital role in achieving better feature extraction results. Here, the value of q has been experimentally set to 0.5. The distributions of textural features provide a statistical basis for identifying healthy and infected lungs from CT scans. Hence, the advantageous effect of the proposed Tq is shown in Fig. 4 in terms of the mean and standard deviation. It can be seen that the features are well clustered into two different classes, which are “healthy” and “infected” and are not overlapping. The well separation of features of the two classes can improve the performance of classification. The logic behind using Tq for feature extraction lies in the capability of Tq to efficiently capture the details of the image according to the pixel’s probability ((x) in Eq. (3)), which represents the texture. This demonstrates the contribution of Tq to this study. Therefore, we can conclude that Tq helps widen the differences between pixel values for the healthy and infected lungs classes.

### Features reduction

In order to optimize the performance of the proposed algorithm, we implemented a feature reduction step to limit the total extracted features to only essential ones. This is done by obtaining the following statistical measures from each extracted feature set: mean, standard deviation, skewness and kurtosis. Feature reduction increases the performance of the algorithm and reduces its overhead and resources allocation, without affecting its classification accuracy. Essentially, for each feature set extracted using the q-transform method (1,024 features for each image), the aforementioned statistical measures are calculated. An optimal set of only 4 features for each image is stored in a one-dimensional array and used as the final feature. This is subsequently used with the classifiers for the classification process.

### Classification

Three types of classifiers were used in this study: “k-nearest neighbor”, “decision tree”, and SVM. These classifiers are accessible from MATLAB R2020b software.

1. k-nearest neighbor. The “k-nearest neighbor” (k-NN) algorithm, as the name suggests, searches for the nearest neighbors in a dataset. “Euclidean function” is often used to calculate the distance, then the values are sorted and assigned to the corresponding class (Nour, Cömert & Polat, 2020). This classifier is one of the most utilized classifiers in the field.
2. Decision tree. The “decision tree” (DT) classifier comprises roots, leaves, and branches which descend from top to bottom. This classifier has been designed for simple classification problems.
3. Support Vector Machine. SVM is a widely used algorithm for classification as well as regression problems. In this algorithm, a pair of data is represented by a point in an n-dimensional space. For binary classification applications (such as the case with our proposed work), a hyper-plane is determined, and then the classification is made. This classifier makes it easy to have a linear hyper-plane between the two classes but the gap between them needs to be optimized (Shafaq et al., 2021).

### Evaluation metrics

Accuracy, specificity and sensitivity were used as the primary evaluation metrics to evaluate the performance of the proposed algorithm, and they are defined as follows:

(4) $$Accuracy = \displaystyle{{TP + TN} \over {TP + TN + FP + FN}}$$

(5) $$Sensitivity = \displaystyle{{TP} \over {TP + FN}}$$

(6) $$Sensitivity = \displaystyle{{TP} \over {TP + FN}}$$

where “TP” (“true positive”) and “TN” (“true negative”) respectively refer to the positive and negative COVID-19 cases (i.e., scans) that are correctly classified as such; while “FP” (“false positive”) and “FN” (“false negative”) represent the number of COVID-19 cases that are incorrectly classified, where a positive case is identified as negative and vice versa. All of the performed operations were carried out using MATLAB 2020b on a system running Microsoft Windows 10.

### EXPERIMENTAL RESULTS AND DISCUSSION

The collected dataset has been used for both training and evaluation purposes. A total of 70% of the scans were used for training, and the remaining 30% were used to evaluate the performance of the algorithm. Five-fold cross-validation has been implemented where the whole dataset was divided into five smaller subsets. Therefore, for each of the iterations, 210 scans were used as training samples, and 90 scans were used for testing. One of the five subsets was used for every iteration. This approach ensures that every CT scan is processed in training and evaluation, thus eliminating any possible bias associated with selective data selection. The code was developed using MATLAB 2020b.

The classification results obtained from the three classifiers (i.e., KNN, Decision tree, and SVM) are presented in Table 2. From Table 2, it can be seen that amongst the three classifiers, SVM performed the best in terms of accuracy, sensitivity, and specificity. Nonetheless, all of the three classifiers produced rather identical rates. This attests to the robustness and flexibility of the proposed feature extraction method.

**Table 2:** Classification results obtained from KNN, Decision tree, and SVM classifiers.

Classifiers	Accuracy %	TP%	TN%	Sens %	Spec %
KNN	98	97.50	98.50	95	97
Decision tree	98	97.60	98.40	95	97
SVM	98.25	97.70	98.80	95.30	97.60

Table 2 shows the results of the COVID-19 classification performance of the proposed method. It can be seen that the detection accuracy is 98.25% with 95.30% sensitivity and 97.60% specificity when using SVM. This shows that using the q-transform method for feature extraction yields an excellent classification accuracy rate. Additionally, it can be noted that the proposed method achieved a rather consistent classification rate (≥98%) for all of the tests, and the classifiers listed in Table 2. This demonstrates that the proposed method works well irrespective of the feature’s complexities of the CT scans.

To further show the strengths of the proposed work, we compared our algorithm with a number of the most popular and recent state-of-art classification methods. Wang et al. (2020) presented a modified CNN-based, transfer-learning model to classify COVID-19 cases through the analysis of lung CT scans. The reported accuracy was 89.5% with specificity and sensitivity of 88% and 87% respectively. Similarly, Wang, Lin & Wong (2020) presented a deep CNN for the classification of COVID-19 cases. The model managed to achieve an accuracy rate of 92.64%, with sensitivity and precision of 91.37% and 95.75%, respectively. As described earlier, Hasan et al. (2020) proposed deep learning and a deformed entropy-based algorithm for the classification of COVID-19 and pneumonia. The algorithm reportedly scored an accuracy rate of 96.20%. Alternatively, Nour, Cömert & Polat (2020) proposed CNN-based feature extraction model which was trained using a novel serial network that comprised five convolution layers. The model achieved an accuracy of 97.14%, with sensitivity and precision rates of 94.61% and 98.29% respectively. In the work presented in Waheed et al. (2020), a new approach to enhance COVID-19 detection accuracy was proposed by developing an “Auxiliary Classifier Generative Adversarial Network” (ACGAN)-based model to generate synthetic chest X-ray images. This approach managed to increase the detection of COVID-19 cases from 85% to 95%.

Comparing these results with Table 3, it is understood that the performance of the proposed classification method is higher. Considering the proposed q-transform model along with feature reduction, it is seen that the proposed q-transform model is more suitable for lung CT scan feature extraction. It can thus be concluded that its effect will be high, especially in the investigation of COVID-19 detection in CT scans.

**Table 3:** Relative comparison with a number of similar, state-of-art methods.

Method	Accuracy %	TP%	TN%	Sens %	Spec %
Wang et al. (2020)	89.50	–	–	87.00	88.00
Wang, Lin & Wong (2020)	92.64	–	–	91.37	95.76
Hasan et al. (2020)	96.20	94.90	98.10	–	–
Nour, Cömert & Polat (2020)	97.14	–	–	94.61	98.29
Waheed et al. (2020)	95	–	–	90	97
Proposed Method (SVM)	98.25	97,70	98.80	95.30	97.60

Although the proposed classification algorithm outperforms the aforementioned algorithms, it should still be taken into account that not all of the algorithms mentioned use the same dataset. Moreover, the proposed method can only differentiate between negative and positive COVID-19 cases from lung CT scans, but cannot discriminate COVID-19 cases from other pneumonia conditions. Nonetheless, due to the proven flexibility of our algorithm, it is highly probable that it can still perform well if tested with different datasets. A summary of the relative comparison is presented in Table 3.

## Conclusion

This study presented a new automated algorithm for the classification of COVID-19 cases from lung CT scans. The algorithm uses a proposed q-transform model along with feature reduction to extract optimal features from the CT scans to ensure an efficient yet accurate performance of the algorithm. The classification was carried out by three different classifiers: the “k-nearest neighbor”, “decision tree”, and the SVM. When tested with a dataset comprising 276 lung CT scans, the algorithm achieved classification accuracy of 98.25%, with 95.30% sensitivity and 97.60% specificity with SVM. These results prove that the proposed method is flexible and efficient, which reflects the strengths of the q-transform model. It is noteworthy that, although feature reduction reduces resources allocation and improves performance, some information from the image may have been lost due to this. Accordingly, this may have affected the classification accuracy, but insignificantly. Possible future improvements include adding the ability to detect other types of pneumonia, and to use other medical imaging formats (e.g., X-ray and MRI scans) as input images while maintaining the same level of efficiency.

## Additional Information and Declarations

### Data Availability

The following information was supplied regarding data availability: Code and CT scan dataset are available at GitHub: https://github.com/hamidjalab/COVID19-qtransform. The full dataset is available at https://www.sirm.org/category/senza-categoria/covid-19/, contact segreteria@sirm.org.

## References

[ref-1] Ahmad F, Farooq A, Ghani MU (2021). Deep ensemble model for classification of novel coronavirus in
chest X-ray images. Computational Intelligence and Neuroscience.

[ref-2] Ahsan M, Based M, Haider J, Kowalski M (2021). COVID-19 detection from chest X-ray images using feature fusion
and deep learning. Sensors.

[ref-3] Al-Shamasneh AaR, Jalab HA, Palaiahnakote S, Obaidellah UH, Ibrahim RW, El-Melegy MT (2018). A new local fractional entropy-based model for kidney MRI image
enhancement. Entropy.

[ref-4] Ala’a R, Jalab HA, Shivakumara P, Ibrahim RW, Obaidellah UH (2020). Kidney segmentation in MR images using active contour model
driven by fractional-based energy minimization. Signal, Image and Video Processing.

[ref-5] Bhattacharya S, Maddikunta PKR, Pham Q-V, Gadekallu TR, Chowdhary CL, Alazab M, Piran MJ (2021). Deep learning and medical image processing for coronavirus
(COVID-19) pandemic: a survey. Sustainable Cities and Society.

[ref-6] Dimitrovski I, Kocev D, Kitanovski I, Loskovska S, Džeroski S (2015). Improved medical image modality classification using a
combination of visual and textual features. Computerized Medical Imaging and Graphics.

[ref-7] Fitouhi A, Bettaibi N (2006). Wavelet transforms in quantum calculus. Journal of Nonlinear Mathematical Physics.

[ref-8] Gadekallu TR, Rajput DS, Reddy MPK, Lakshmanna K, Bhattacharya S, Singh S, Jolfaei A, Alazab M (2020). A novel PCA-whale optimization-based deep neural network model
for classification of tomato plant diseases using GPU. Journal of Real-Time Image Processing.

[ref-9] Hasan AM, Al-Jawad MM, Jalab HA, Shaiba H, Ibrahim RW, AL-Shamasneh Aa R (2020). Classification of covid-19 coronavirus, pneumonia and healthy
lungs in CT scans using q-deformed entropy and deep learning
features. Entropy.

[ref-10] Hasan AM, Jalab HA, Meziane F, Kahtan H, Al-Ahmad AS (2019). Combining deep and handcrafted image features for MRI brain scan
classification. IEEE Access.

[ref-11] Ibrahim RW, Hasan AM, Jalab HA (2018). A new deformable model based on fractional Wright energy function
for tumor segmentation of volumetric brain MRI scans. Computer Methods and Programs in Biomedicine.

[ref-12] Jalab HA, Ibrahim RW, Ahmed A (2017). Image denoising algorithm based on the convolution of fractional
Tsallis entropy with the Riesz fractional derivative. Neural Computing and Applications.

[ref-13] Jalab HA, Subramaniam T, Ibrahim RW, Kahtan H, Noor NFM (2019). New texture descriptor based on modified fractional entropy for
digital image splicing forgery detection. Entropy.

[ref-14] Kumar A, Kim J, Lyndon D, Fulham M, Feng D (2016). An ensemble of fine-tuned convolutional neural networks for
medical image classification. IEEE Journal of Biomedical and Health Informatics.

[ref-15] Li L, Qin L, Xu Z, Yin Y, Wang X, Kong B, Bai J, Lu Y, Fang Z, Song Q, Cao K, Liu D, Wang G, Xu Q, Fang X, Zhang S, Xia J, Xia J (2020). Using artificial intelligence to detect COVID-19 and
community-acquired pneumonia based on pulmonary CT: evaluation of the
diagnostic accuracy. Radiology.

[ref-16] Nour M, Cömert Z, Polat K (2020). A novel medical diagnosis model for COVID-19 infection detection
based on deep features and Bayesian optimization. Applied Soft Computing.

[ref-17] Olayemi AD, Zare MR, Fermi PM (2019). Medical image classification: a comparison of various handcrafted
features. journal of Advances in Soft Computing and its Applications.

[ref-18] Öztürk Ş, Özkaya U, Barstuğan M (2021). Classification of Coronavirus (COVID-19) from X-ray and CT images
using shrunken features. International Journal of Imaging Systems and Technology.

[ref-19] Roy S, Shivakumara P, Jalab HA, Ibrahim RW, Pal U, Lu T (2016). Fractional poisson enhancement model for text detection and
recognition in video frames. Pattern Recognition.

[ref-20] Shafaq A, Jalil Z, Javed AR, Batool I, Khan MZ, Noorwali A, Gadekallu TR, Akbar A (2021). BCD-WERT: a novel approach for breast cancer detection using
whale optimization based efficient features and extremely randomized tree
algorithm. PeerJ Computer Science.

[ref-21] Shankar K, Perumal E (2020). A novel hand-crafted with deep learning features based fusion
model for COVID-19 diagnosis and classification using chest X-ray
images. Complex & Intelligent Systems.

[ref-22] Song Y, Zheng S, Li L, Zhang X, Zhang X, Huang Z, Chen J, Zhao H, Wang R, Chong Y, Shen J, Zha Y, Yang Y (2020). Deep learning enables accurate diagnosis of novel coronavirus
(COVID-19) with CT images. MedRxiv.

[ref-23] Umarov S, Tsallis C, Steinberg S (2008). On aq-central limit theorem consistent with nonextensive
statistical mechanics. Milan Journal of Mathematics.

[ref-24] Vasan D, Alazab M, Wassan S, Naeem H, Safaei B, Zheng Q (2020). IMCFN: image-based malware classification using fine-tuned
convolutional neural network architecture. Computer Networks.

[ref-25] Venkatraman S, Alazab M (2018). Use of data visualisation for zero-day malware
detection. Security and Communication Networks.

[ref-26] Waheed A, Goyal M, Gupta D, Khanna A, Al-Turjman F, Pinheiro PR (2020). Covidgan: data augmentation using auxiliary classifier GAN for
improved covid-19 detection. IEEE Access.

[ref-27] Wang L, Lin ZQ, Wong A (2020). Covid-net: a tailored deep convolutional neural network design
for detection of covid-19 cases from chest x-ray images. Scientific Reports.

[ref-28] Wang S, Kang B, Ma J, Zeng X, Xiao M, Guo J, Cai M, Yang J, Li Y, Meng X, Xu B (2020). A deep learning algorithm using CT images to screen for Corona
Virus Disease (COVID-19). MedRxiv.

[ref-30] World Health Organization (2021). Coronavirus disease (COVID-19) pandemic. https://www.who.int/emergencies/diseases/novel-coronavirus-2019.

[ref-29] Zhang W, Pogorelsky B, Lovelandy M, Wolf T (2021). Classification of COVID-19 X-ray images using a combination of
deep and handcrafted features. http://arxiv.org/abs/2101.07866.

